# Literary Gossip and Record

**Published:** 1861-10

**Authors:** 


### ART. XIII.—LITERARY GOSSIP AND RECORD.

IF we glance back at the past twelve months and seek to estimate the merit of the period by its literary productiveness, the result is not very exhilarating. If we look forward and endeavour to ascertain the promise of the forthcoming year, the prospect is by no means encouraging.

Not that the past twelve months have been altogether wanting in notable works, or that the future twelve are entirely devoid of promise; or that the numerical value of the works published in the former period or promised in the latter is more than ordinarily insignificant; but a preponderance of minor works marks the one, and would appear to be threatened by the other.

Yet it is to be remarked, that the literary deadness of the past year was not peculiar to this country. In France, little literary vitality seems also to have been manifested during the same time.

If, however, the literary retrospect is not very exhilarating, it is by no means wanting in interest. There was a demand for new editions of recent and comparatively recent works, which must have been most gratifying to the authors, and which told well for the worth and stability of many of the additions to English medical literature within the last three or four years. Many new editions of recent works also characterize the promise for the coming year.

Of the new works published since the 1st of October, 1860, the chief place in literary importance must be assigned to Dr. Maynes' *Expository Lexicon of the Terms, Ancient and Modern, in Medical and General Science;* in physiological, to Dr. Edward Smith's *Treatise on Health and Disease, as influenced by the Daily, Seasonal, and other Cyclical Changes in the Human System;* in pathological, to Dr. Chance's translation of Virchow's *Cellular Pathology.* John Hunter's *Essays and Observations on Natural History, Anatomy, Physiology, Psychology, and Geology,* edited by Mr. Owen, have a special interest apart. The publication of the *Roll of the College of Physicians* is a happy and valuable addition to a portion of our medical literature too much neglected; and Dr. Meryon's *History of Medicine,* when completed, will supply a want much felt.

Mr. Holmes's *System of Surgery* will successfully fill up a great hiatus in our encyclopædical literature; and Mr. Barwell's excellent treatise on *Diseases of the Joints* will take its place among standard works on surgery.

In medicine we have the works of Dr. Parker and Dr. Beale on the *Urine;* Dr. Lyons on *Fever;* Drs. Jenner and Greenhow on *Diphtheria*—the latter a valuable and complete treatise, the former a brief practical dissertation on the subject; Dr. Copland on *Consumption and Bronchitis;* Dr. Goodfellow on *Bright's Disease;* and Dr. Routh on *Infant Feeding*.

Finally, we would note two interesting additions to medical biography—the *Autobiography of Sir James Macgregor,* and the *Life of Marshall Hall,* by his widow.

This is certainly a most scanty list, but if the quantity is defective, the quality is by no means to be complained of.

Of the works promised in the present quarter, we shall direct attention to one only—a work on *Hygiene,* designed especially for the use of officers of health, by Drs. Lankester and Letheby. This will be a most welcome addition to medical literature: would that the same gentlemen were about to give us a complete treatise on the subject! It is a curious and most inexplicable fact, that notwithstanding the unusual amount of attention which has been given practically to Hygiene, both public and private, in this kingdom during the last twenty years, not a single complete treatise, not even a manual of the subject, exists in the English language.

